# The Northern Ireland Control Programmes for Infectious Cattle Diseases Not Regulated by the EU

**DOI:** 10.3389/fvets.2021.694197

**Published:** 2021-08-26

**Authors:** Sam Strain, Sharon Verner, Emma Campbell, Jaka Jakob Hodnik, I. M. G. A. Santman-Berends

**Affiliations:** ^1^Animal Health and Welfare Northern Ireland, Dungannon, United Kingdom; ^2^Veterinary Sciences Division, Disease Surveillance and Investigation Branch, Agri-Food and Biosciences Institute, Belfast, United Kingdom; ^3^Clinic for Reproduction and Large Animals—Section for Ruminants, Veterinary Faculty, University of Ljubljana, Ljubljana, Slovenia; ^4^Royal GD, Deventer, Netherlands

**Keywords:** Northern Ireland, control programmes, cattle, disease, non-regulated, BVD

## Abstract

The disease control programmes for Bovine Viral Diarrhoea (BVD), Infectious Bovine Rhinotracheitis (IBR), Johne's Disease (JD), Leptospirosis and Neosporosis are described including the approved diagnostic tools, diagnostic quality systems, and the role of vaccination (where appropriate). This paper describes the control programmes within NI, the challenges relating them, as well as assessing their impact and effectiveness, taking into consideration the quality of data available and number of herds participating. With the NI agricultural industry experiencing increasing financial pressures and post Brexit changes, the necessity of working to maximise the performance of bovine disease control programmes at the individual farm level as well as at the regional level is increasingly important. The programmes described fall into two categories with two distinct aims. Two managed by Animal Health & Welfare NI (AHWNI), the BVD eradication and JD Dairy Control programmes seek to eradicate or control infection at the regional level. A further 5 programmes, covering BVD, JD, IBR, Leptospirosis and Neosporosis, are managed by the Agri-Food and Biosciences Institute (AFBI) and focus on facilitating eradication or control at the individual herd level. These latter programmes conform to the Cattle Health Certification Standards (UK) (CHeCS) which is a UK self-regulatory body set up to ensure consistency between different disease control schemes across herds. The largest of all the programmes described is the AHWNI BVD Eradication Programme which has led to significant reductions in infection incidence. Compliance with it has been high with more than 97% of all cattle alive at the end of 2020 having a BVD test status. The rolling annual incidence of BVD virus positive calves has fallen by 56% since the start of the compulsory programme in 2016. This decrease has occurred largely through industry initiatives to deal with BVD positives, including the voluntary culling of persistently infected (PI) animals by herd owners, a voluntary abattoir ban on the slaughter of BVD virus (BVDv) positive animals, and the inclusion of retention of a BVDv positive animal as a non-conformance in the industry-run Farm Quality Assurance Scheme.

## Introduction

There are ~1.6 million cattle on ~22,000 farms in Northern Ireland (NI). Of these approximately 20,000 have breeding cows, with approximately 2,600 of these herds being primarily dairy. The average number of cows per dairy herd is 95 and per beef herd is 17 (1). Each year there are over 500,000 calf births registered. Given that there is ~1 million hectares farmed within Northern Ireland, this means the region has the highest cattle density within the UK and is amongst the highest across Europe (2). Importantly there are very substantial numbers of intra and inter herd animal movements leading to a high level of interconnectedness between herds. The result of this is substantial vulnerability to pathogen spread between herds (3, 4). There are also substantial risks to infection spread between holdings due to the fragmented nature of farms within NI leading to multiple potential points of contact between grazing herds (5). The calving pattern is somewhat seasonal with a peak in April and May. However, there are also substantial numbers of calvings at all other points in the year. Therefore, for reproductive diseases such as BVD, there are susceptible pregnancies present all year round within the region.

The NI cattle industry including Dairy Council for Northern Ireland, the Northern Ireland Meat Exporters Association, the Ulster Farmers' Union and the Northern Ireland Agricultural Producers' Association as well as the Association of Veterinary Surgeons Practising in Northern Ireland have been the key drivers behind the development and implementation of significant and innovative programmes designed to control endemic infectious diseases of cattle that have not been subject to mandatory EU regulation. The context for these programmes is a trend toward a shared responsibility for animal health policies and costs between the agri-food industry and government. Increasingly there is a requirement for the NI industry to provide leadership and influence priority-setting in the control of endemic diseases. This paper describes the current control programmes within NI and sets out the management framework for them.

NI has implemented a number of disease control programmes. Animal Health and Welfare NI (AHWNI) manages two programmes that aim to eradicate BVD or control Johne's Disease across Northern Ireland. The Agri-Food and Biosciences Institute (AFBI) manages a further 5 voluntary disease control programmes as part of its Cattle Health Scheme (CHS) covering BVD, IBR, JD, Neosporosis, and Leptospirosis which focus more on assisting individual herds to control or eradicate infection.

AHWNI is a not-for-profit organisation formed by industry and mandated to lead on the co-ordination of the control of non-regulated endemic diseases. The AHWNI BVD eradication programme is unique in the UK. It is overseen by an Implementation Group which is chaired by AHWNI and comprises a range of stakeholders, including practising veterinary surgeons, farming unions, breed society representatives, the NI Farm Quality Assurance Scheme organisation, Animal Health Ireland veterinarians and, as observers, members of the Department of Agriculture, Environment and Rural Affairs (DAERA). As well as this, AHWNI manages a recently launched JD control programme for dairy herds.

AFBI is a non-departmental public body providing research and development, diagnostic and analytical testing for government and commercial companies in Northern Ireland. It launched its Cattle Health Scheme in 2008. These programmes are licensed by and comply with the UK body Cattle Health Certification Standards (CHeCs).

The purpose of this paper is to describe all of these programmes, their findings and in the case of the BVD eradication programme, progress toward eradication of this disease.

## Materials and Methods

### Data Sources and Analysis

Summary anonymised data was sourced from the Department of Environment, Agriculture and Rural Affair's (DAERA) Animal and Public Health Information System (APHIS), Animal Health and Welfare NI's (AHWNI) BVD database, and the Agri-Food and Biosciences Institute's (AFBI) Veterinary Sciences Division. Data was visualised and analysed using GraphPad Prism 9.1, and maps were generated using QGIS 3.6.2 (GNU Public License).

### Management & Coordination of Control Programmes

The control programmes reviewed in this paper are managed by either AHWNI or the AFBI.

The technical aspects of the AHWNI BVD eradication and Johne's Dairy Control programmes were designed using the technical expertise of all-island (Northern Ireland and Republic of Ireland) technical working groups (TWGs). The TWGs draws on a range of expertise including from veterinarians with a special interest in the respective infections as well as laboratory experts and academics. The operation of BVD programme is overseen by a local Implementation Group (IG) composed of representatives from across the NI cattle producer and processor industry.

AFBI also offers five voluntary programmes covering BVD, IBR, JD, Neosporosis, and Leptospirosis for cattle farmers within NI. These schemes are licensed by the UK body Cattle Health Certification Standards (CHeCs), which was formed in 1999 by stakeholders across the cattle industry in the UK. The aim of CHeCs licensed programmes is to provide a protocol for controlling and eliminating infectious endemic diseases in cattle at farm or herd level. The Standards set out the required protocols including testing requirements as well as biosecurity recommendations and requirements (6). The AFBI Cattle Health Scheme (CHS) was established in 2008.

### Control Programme Descriptions

#### AHWNI BVD Eradication Programme

The overarching aim of the BVD Eradication Programme is to eradicate the infection from NI. It is built on the following principles:

- Testing of all new-born calves including those stillborn for BVD virus (BVDv) RNA or antigen.
- Identification of cattle with non-negative BVDv results and isolation of high infectious risk animals.
- Improving stakeholder knowledge of BVD and awareness of biosecurity principles through a continuous flow of information.
- Private veterinary practitioner involvement through the provision of herd test information, advice to herd owners and follow-up testing.
- Restrictions on the movement of non-negative animals.
- Voluntary removal of BVDv Persistently Infected (PI) cattle.

Underpinning the programme is the AHWNI BVD database which collates animal data from DAERA's APHIS system, animal identification tag sales from approved tag suppliers and test results from approved laboratories as well as generating automated Short Message Service (SMS) text messages and farmer information letters. BVD statuses are automatically ascribed to animals including indirect statuses to the dams of tested calves and the statuses are uploaded to DAERA's APHIS database.

Laboratory tests for use by the programme must be approved by DAERA following advice from AHWNI on their suitability. Tests must be able to detect BVD virus via ELISA antigen or by PCR, the kits used must be approved by the Friedrich Loeffler Institute, and the tests must be carried out to the ISO 17025 standard. Blood samples from calves under 75 days of age may not be reliably tested by ELISA antigen testing due to the possibility of false negative results caused by interference from maternally derived antibodies. For this reason, negative results from such animals are considered valid only if produced by a RT-PCR test. Further details of the approval criteria are contained in the Supplementary Material.

A voluntary phase of the NI BVD Eradication Programme commenced on January 1, 2013. The aims of the programme were to identify PI animals through the testing of ear tissue samples collected at tagging for identification of new-born calves as well as generating foundational knowledge and experience for the later compulsory phase of the programme. The compulsory phase started on March 1, 2016 with the introduction of supporting legislation, The Bovine Viral Diarrhoea Eradication Scheme Order (Northern Ireland) 2016 (7). This order enshrines in law the programme as described for the voluntary phase. It requires the keeper of cattle to take a tissue sample for analysis for BVDv from new-born calves, aborted foetuses, stillborn calves, and calves which have died before tagging as well as any bovines born after March 1, 2016 which come into the possession of a keeper and do not possess a negative test result for BVDv. Samples must be posted to designated laboratories (approved by DAERA) within 7 days of sample collection. Repeat analysis of cattle with non-negative test results and inadequate tissue samples is provided for. BVDv Negative Status may be allocated to a bovine animal where a test is negative for BVDv. Any animal with a positive BVDv test result is given a BVD positive (BVDP) status. A keeper can choose to undertake follow-up testing carried out by a veterinarian of BVDP animals to differentiate persistently infected animals from transiently infected ones. Where there is a negative follow-up BVDv test, the animal's status is set as negative. For the purposes of the programme all animals with non-negative BVD statuses are restricted from moving to other herds. In addition, keepers are required to isolate infectious or potentially infectious bovines and follow-up testing of bovines suspected of being infected with BVDv, such as the dams of BVDP calves is recommended.

Ear tissue samples can be analysed using antigen-capture ELISA or RT-PCR methods. For blood samples from calves up to and including 75 days of age, the RT-PCR test is used; blood samples from older calves may be tested using RT-PCR or antigen ELISA. Retested animals with a BVDv negative result are considered as having been transiently infected and an indirect negative status is applied to their dam. Where a calf tests positive, the dam is categorised as being suspect and a “Dam of a PI” (DAMPI) status is applied in the absence of a direct negative status for the dam. Such animals can be follow-up tested for BVDv by blood sample taken by a veterinary practitioner. A negative test result will allow the animal to be assigned a test negative status. A description of the statuses used in the programme are listed in Table 1.

**Table 1:** Description of the BVD statuses used within the AHWNI BVD eradication programme.

**BVD status**	**Description**	**Are off farm movements permitted?**
BVDN	Animal has had a direct negative test result	Yes
INDNEG	Animal is the dam of a BVDN calf so can be given indirect negative status	Yes
“Blank”	Animal born before 01/03/16 where the BVD status is unknown	Yes
BVDP	Animal has had a direct positive test result	No
BVDI	Animal has had a direct inconclusive test result	No
DAMPI	Animal is the dam of a BVDP or BVDI calf	No
OFFPI	Offspring of a BVDP dam	No
BVDU	Animal born since 01/03/16 where the BVD status is unknown—either because it has not been tested or the sample was inadequate.	No

The AHWNI BVD Programme has focused on providing prompt and targeted communications to stakeholders in the programme, particularly herd owners in receipt of non-negative results. Social media is used to pass on key messages and provide statistical updates to advertise progress (@animalhealthni). Where a herdowner has supplied a mobile number, all results are communicated via a SMS text message. All herds with non-negative test results also receive notification and advisory letters. Where there are long-standing herds with untested or positive animals the AHWNI secretariat follows up with further SMS text messages and advisory phone calls.

Herdowners are able to nominate a private veterinary practitioner to receive results for their clients' herds, and to facilitate follow-up testing, herd investigation and the provision of biosecurity advice.

Laboratories report results to the AHWNI database, which records all BVD test results against the animal identifications and applies BVD statuses which are then uploaded from the AHWNI database to the APHIS system. An Indirect Negative status is applied to the dams of BVD Negative calves, and positive (DAMPI) statuses are applied to untested dams of PIs and their offspring. The BVD statuses on APHIS and the BVD database may be viewed by the current herd owner. Movement restrictions are applied on APHIS to all cattle born in the compulsory programme period that have non-negative results and to untested dams of PIs and their offspring until they are in possession of a BVD Negative test result.

BVD test results are notified to farmers by SMS text messages to their nominated mobile telephone numbers. When a non-negative result is returned, in addition to SMS texts, a letter is issued to the herd owner and their nominated veterinary practitioner is informed of the results for their clients' herds. Immediate isolation in housing is required by law and potentially subject to enforcement by DAERA. The herd owner has the option to retest the animal 3 weeks after the initial sample was taken, using a blood sample taken by a private veterinarian.

BVD is not a notifiable disease in NI and vaccination is allowed. Vaccination does not currently interfere with the eradication programme as it is based upon the detection of BVDv. All bulls licensed for artificial insemination in NI are tested for BVDv.

A key challenge to the programme is the timely removal of persistently infected animals. There are no support public support payments available for the removal of BVDv positive animals. To encourage the disposal of these animals, the NI industry has unilaterally put two voluntary measures into place. The first is a voluntary abattoir ban on the slaughter of BVD positive animals. This was an initiative made by all the major abattoirs to agree to refuse for slaughter any animal born during the compulsory phase of the programme that has a positive BVDv test result. The purpose of this was to support the eradication programme by removing any incentive herdowners might have to retain PI animals in the hope that they could be finished as beef animals. The second was the inclusion of retention of a BVD positive animal as a non-conformance in the industry-run Farm Quality Assurance Scheme (FQAS) for beef animals. In this case any member of the FQAS in the possession of a bovine with a positive test result for BVDv will have their farm quality attained status removed from the herd if the BVD status of the bovine in question is not resolved. The status can be resolved either through evidence that a BVD negative test result has been obtained for the animal or through evidence that the animal has been culled or died. Within NI the majority of beef animals are included in the FQAS scheme, as there can be considerable financial penalties for animals slaughtered that are not FQAS assured^1^. Both these measures were voluntarily negotiated with the NI industry to disincentive herdowners from retaining PI animals and therefore assist with reducing the transmission of infection within and between herds.

#### AHWNI Johne's Disease Control Programme for Dairy Herds

The voluntary AHWNI Johne's Disease Control Programme (JDCP) for dairy herds was launched in October 2020. The objectives of the programme are to provide herdowners with all available tools and information to support a robust JD control programme in NI. The design of the programme is in line with the international experience of Johne's Disease control programmes (8). The key goals of the programme are:

- **Bio-exclusion**. To help identify those herds that test negative for Johne's disease and provide these farmers with the knowledge and professional support to allow them to increase their confidence over time of being free of infection and to protect their herds from the on-going risk of introduction of this infection.
- **Bio-containment**. To provide herds identified as being infected or having a low confidence of freedom from infection, with the knowledge and professional support to allow them to control and reduce the prevalence of the disease over time and ultimately to achieve a high confidence of freedom from infection for those herds wishing to progress to this level.
- **Market reassurance**. To underpin the quality of Northern Irish animal produce in the national and international marketplace.

The required components for participating in the programme are:

- Programme enrolment including acceptance of the programme's Terms & Conditions.
- The provision by an Approved Veterinary Practitioner of a Veterinary Risk Assessment and Management Plan (VRAMP).
- Electronic uploading to AHWNI of VRAMP findings and recommendations.
- Limitation on the sale of JD positive/inconclusive animals.

In addition to the mandatory components, it is strongly advised that participating herds undertake whole herd testing for the infection. All animals in the herd over 2 years of age should be tested and the herd screen should be completed within 12 months of enrolment or within 12 months of the previous herd screen. Currently the two tests that are recommended for herd screening within the AHWNI JDCP are individual animal milk and blood ELISA tests. In addition, two tests are recommended as ancillary tests, individual animal faecal culture or PCR. AHWNI currently recommends that all tests are carried out to the ISO 17025 standard and that only those kits that are approved by the Friedrich Loeffler Institute are used.

The VRAMP is a detailed on-farm review carried out annually by an approved veterinary practitioner (AVP) in partnership with the farmer to identify aspects of management that could predispose to the introduction (Bio-exclusion) and spread of infection within the farm (Bio-containment) and to provide recommendations for the reduction of these risks.

The VRAMP uses a scoring system which assists the identification of high-risk practices and areas within the farm on which control should be focussed. It focuses on:

- Infection history, that is, clinical and test evidence of historical infection.
- Biosecurity risks, for example, animal moves and mixing with other herds and the risk of exposure to faecal material from other herds.
- Pre-weaned calf risks, for example, sources of milk, feeding regimes, group rearing practices and exposure to adult faeces.
- Young stock cleanliness including exposure to adult faeces.
- Calving risks, for example, cow cleanliness, risk of calf exposure to multiple cows and management of high-risk cows such as those with positive JD test results

As a consequence of the assessment, up to three agreed farm-specific practical recommendations are made at each assessment visit to reduce infection risk that both the farmer and the AVP agree can be implemented on the farm. Only veterinary practitioners who have undergone specific training provided by AHWNI can be approved by AHWNI to undertake the assessments.

After herds have completed an initial VRAMP a follow-up risk assessment should be carried out during every subsequent 12-month period. These follow-on assessments are essential to monitor progress that the herd may have made in mitigating JD related risks. This allows an assessment of how successfully the management plan has been carried out so that changes in recommendations can be made where necessary and new emerging risks can be identified.

To facilitate the carrying out of the VRAMP, AHWNI has developed an online tool which can be accessed online using a smartphone (https://ahwni.wufoo.com/forms/veterinary-risk-assessment-and-management-plan/). The purpose of this is to assist with the carrying out, recording, and uploading of the VRAMP in real time on farm. Where the online portal cannot be accessed the VRAMP can be completed by hard copy. However, to comply with the programme all findings must be uploaded to AHWNI.

### AFBI Cattle Health Scheme Programmes

The diseases covered by the AFBI CHS are JD, BVD, IBR, Leptospirosis and Neosporosis. Herds that meet the standards of each disease programme can gain herd accreditation for that disease. The CHeCs technical document (9) outlines the requirements of each party (farmer, private veterinary practitioner (PVP) and laboratory) in meeting the standards of accreditation for the disease programmes.

The CHS BVD and JD programmes are complimentary to the AHWNI BVD and JD programmes. For example, the testing carried out for the AHWNI BVD eradication programme can be used for the CHS BVD programme. However, depending on the CHS programme followed, farmers may be required to adopt additional biosecurity measures in order to comply with CHS rules (see below). The CHS for JD, while available for all herd types, has been adopted mostly by pedigree herds, particularly pedigree beef herds. Therefore, it provides a valuable compliment to the AHWNI JD programme for dairy herds, for example through the identification of low-risk stock bulls for purchase by dairy herds.

Farmers are required to follow the CHeCs rules regarding biosecurity, added/returning animal testing, isolation requirements and ensuring all eligible animals are tested annually. The farm's PVP is instrumental in supporting the farmer in achieving and maintaining their disease programme statuses. PVPs may offer advice regarding what programmes to participate in, vaccination (if required) and advice in the event of a breakdown of a disease. The PVP is required to inspect the herd and take the appropriate samples. A submission form signed by the farmer and PVP certifying that they are following the rules applicable to them as outlined in the CHeCs technical document is required when submitting samples (9). A listing of vaccines currently available within the UK can be found at https://www.vmd.defra.gov.uk/ProductInformationDatabase/.

While specific test kits are not prescribed within CHeCs approved schemes all testing involved must be carried out under the United Kingdom Accreditation Service (UKAS) ISO/IEC 17025 standards with each method completing the appropriate Quality Assurance scheme testing.

#### Johne's Disease

Due to the limited sensitivity of the tests for Johne's Disease (10) the AFBI Johne's CHS programme works by awarding herds a risk level status rather than an accredited free status depending on testing results (Table 2). As part of the CHeCs rules animals confirmed to be shedding *Mycobacterium avium* subspecies *paratuberculosis* (MAP) must be culled and their last registered progeny should not be retained or sold on for breeding.

**Table 2:** Summary of Johne's risk level criteria.

**Risk level**	**Definition**
Risk Level 1	Herds must have had three consecutive clear herd tests at annual intervals. Level 1 will be further defined by stating the year in which the herd achieved level 1 assessment. This is associated with the lowest risk of Johne's disease in relation to buying breeding stock from participating herds.
Risk Level 2	This applies to all herds that have had an initial, or two consecutive clear tests, but are yet to achieve level 1 status. Level 2 will be further qualified by the number of consecutive clear herd tests that have been achieved (e.g., Level 2, 1 year clear; Level 2, 2 years clear).
Risk Level 3	These are herds that have test positive animals identified within the herd, but the number of test positive animals does not exceed 3% of the herd eligible for testing in the Johne's programme at the most recent test.
Risk Level 4	These herds have more than 3% of eligible animals identified as test positive animals at the most recent test.
Risk Level 5	These herds may be carrying out a testing programme but are not adhering to the mandatory requirements of the programme.

All animals over 2 years of age must be tested for Johne's Disease antibodies annually. Any animal that tests positive or inconclusive requires follow-up testing. For inconclusive animals, they may have to repeat antibody testing performed 30 days after the initial sample or a faecal sample submitted for MAP PCR testing. Animals that test positive on serology can only have follow-up MAP PCR testing performed. A positive MAP PCR result confirms MAP within the herd. As well as this annual herd test, all added animals are required to be tested for MAP antibodies by serology testing and to have faeces tested for MAP by PCR. Both results have to be negative, or the animal cannot join the herd. If an animal returns to the herd after 7 days of being away, it also must be tested for MAP antibodies and to have faeces tested for MAP by PCR, and again both are required to be negative.

#### BVD

There are two AFBI Cattle Health Scheme BVD programmes, an Accredited Free (AF) programme and the Vaccinated Monitored Free (VMF) programme. The AF programme has a superior status to the VMF programme and requires herds to have 3 m double fencing around the entire farm boundary. Due to issues with costs and land space, not all farmers can achieve double fencing of their farm. To allow farmers who would like to achieve a BVD status but cannot double fence their farms there is the option to join the VMF programme. The BVD VMF programme requires vaccination of the breeding herd but does not require 3 m double fencing to be in place. Due to the AHWNI BVD eradication programme in NI described above, farmers can use their statutory ear notch testing results for use in their annual BVD herd test at no additional expense. To achieve BVD AF or VMF status, herds must have 2 years' negative results as well as follow the programme rules. All added animals require BVD antibody and antigen testing after being in quarantine for at least 28 days. Animals <75 days old are required to have BVD antigen testing performed by PCR to avoid the interference of maternally derived antibodies. Animals over 75 days can be tested by BVD antigen ELISA. Depending on the results, animals may be allowed to enter the main herd, have further testing performed or remain in quarantine until they have calved. Animals with a positive antibody result can enter the main herd after the 28-day quarantining period, however pregnant animals should remain in quarantine until they have calved, and the calf is known to be negative for BVD virus. An exception to this is allowed if the animal was known to be BVD antibody positive or vaccinated prior to service. Members are also warned that there is a small risk that BVD antibody positive bulls can excrete BVD virus in semen for several months after infection (11).

#### IBR

The IBR programme offers two options, the Accredited Free (AF) or the Vaccinated Monitored Free (VMF) options with the same provision regarding double fencing as for the AF BVD programmes. However, since the conventional/wild type IBR vaccine is licensed in NI (12) it is a requirement that animals receiving an IBR vaccine are given an IBR marker vaccine to enable vaccinated animals to be differentiated from animals with natural infection. Some animals may be exempt from the vaccination protocol on the farm if appropriate, for example, a breeding bull that may be sent to an artificial insemination (AI) station. To achieve either the IBR AF or VMF status, herds are required to have two consecutive negative herd tests including all animals over 1 year old for IBR antibodies. These two qualifying herd tests can be performed 1–12 months apart. Once the status has been achieved annual herd testing is carried out on all animals over 1 year old. A positive IBR antibody result in a herd test is classified as a failed annual herd test and the herd's status is suspended until the herd can achieve two further qualifying herd tests. Added and returning animals are required to be quarantined and tested for antibody at least 28 days after entering quarantine facilities on the farm. When a subsequent negative test result is available, the animal is allowed to enter the farm.

For herds which are using a marker vaccine in their herd the gE deleted antibody ELISA test is used, whereas for herds not using a vaccine the whole virus antibody ELISA test is used instead.

#### Leptospirosis

The Leptospirosis programme addresses *Leptospira* Hardjo and does not allow for vaccination within participating herds as the diagnostic test cannot differentiate between exposure and vaccination. Any herd considering starting/stopping Leptospirosis vaccination is advised to consult with their PVP. The Leptospirosis programme has two options: Accredited Free (AF) and Monitored Free (MF). The Leptospirosis AF status applies where the herd is free from Leptospirosis infection and all animals test negative for antibodies. The Leptospirosis MF programme can be awarded despite the presence of a small number of test positive animals in the herd (a single test positive animal in herds with 20 or fewer breeding animals, or up to 5% of breeding animals in larger herds), and where there is no evidence of disease transmission. To achieve either status, two herd tests are required 6–12 months apart. All animals 2 years and older, plus any females or males between 1 and 2 years of age which are intended for breeding must be tested. Once either status has been achieved annual herd testing is required as well as testing all added and returning animals after 28 days in quarantine.

Given the zoonotic risk of leptospirosis one component of the programme is to highlight to farmers the risk of infection and their responsibilities under UK law, specifically the Control of Substances Hazardous to Health (COSHH) regulations, to protect themselves and their employees.

#### Neospora

The Neosporosis programme applies Risk Levels rather than an Accredited Free status. The definition of each Risk Level is shown in Table 3. At each annual herd test all female animals aged 2 years and older, plus any females between 1 and 2 years of age which are intended for breeding must be tested including any added female animals after arrival for Neospora antibodies.

**Table 3:** Summary of neosporosis risk level criteria.

**Risk level**	**Definition**
Risk level 1	Herds must have had three consecutive clear annual herd screens. Level 1 will be further defined by stating the year in which the herd achieved level 1 assessment. This is associated with the lowest risk of neosporosis in relation to buying breeding stock from participating herds.
Risk level 2	This applies to all herds that have had an initial, or two consecutive clear tests, but are yet to achieve level 1 status. Level 2 will be further qualified by the number of consecutive clear herd tests that have been achieved (e.g., Level 2, 1 year clear; Level 2, 2 years clear).
Risk level 3	These are herds that have test positive animals identified within the herd, but the number of test positive animals does not exceed 5% of the herd eligible for testing in the Neosporosis programme at the most recent test.
Risk level 4	These herds have more than 5% of eligible animals identified as test positive animals at the most recent test.
Risk level 5	These herds may be carrying out a testing programme but are not adhering to the mandatory requirements of the programme.

## Results

### BVD AHWNI Eradication Programme

426,543 animals were tested in 4,519 herds (~23% of breeding herds) during the voluntary phase of the Programme (January 2013 to March 2016). Of these animals, 3396 (0.80%) returned a positive BVDv result. 833 (18.4%) of the participating herds had at least one test positive animal. During 2015, 175,356 animals were tested which was 37% of all animals <1 year of age (13).

During the compulsory phase, overall, herd owner engagement and compliance with the programme has been high with 97.74% of all cattle alive having an ascribed BVD status (Dec 2020). As testing of calves is compulsory, all breeding herds are required to participate in the programme. In total 20,408 herds have participated in the programme for the period up to the end of 2020. 75.1% of herdowners have given permission for BVD results to be shared with a nominated veterinary practice. Since the commencement of the compulsory programme up to the end of 2020, 506,415 SMS text messages had been sent to farmers informing them of their results as well as other programme related information.

**Figure 1:**
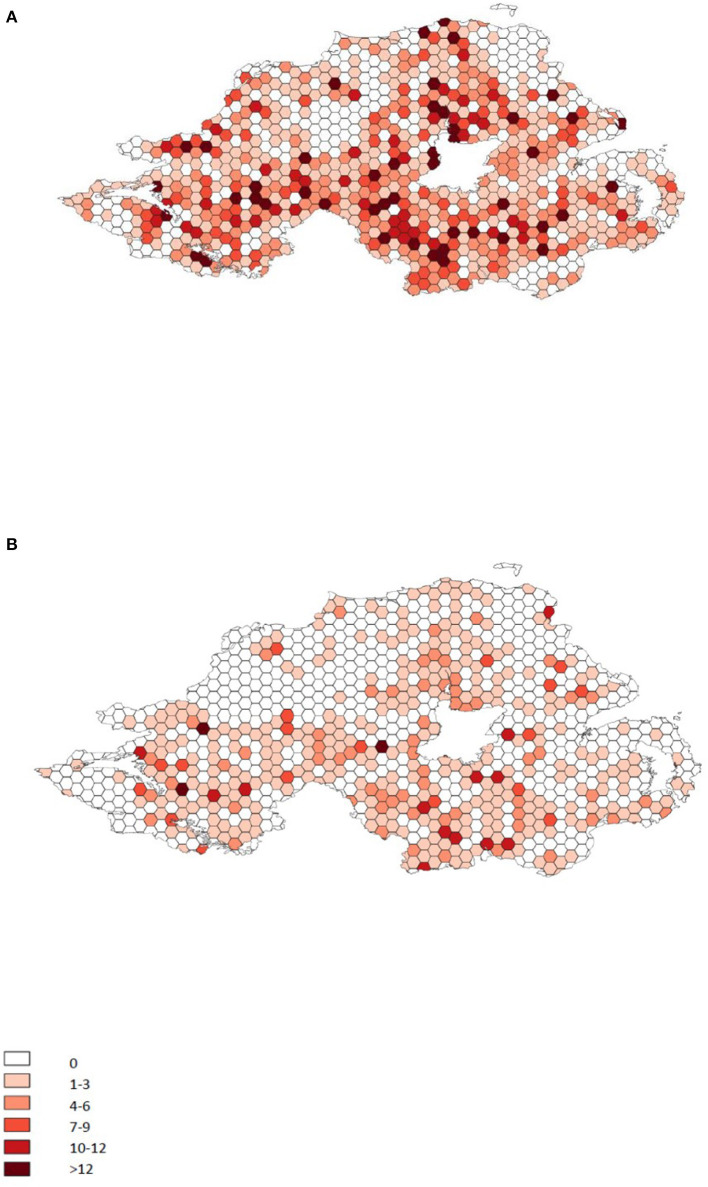
The distribution of disclosed infection across Northern Ireland during 2017 **(A)** and 2020 **(B)**. Hexagons represent an area of ~15 km^2^. Colours represent the number of animals disclosed as BVDP in that area for each year.

**Figure 2:**
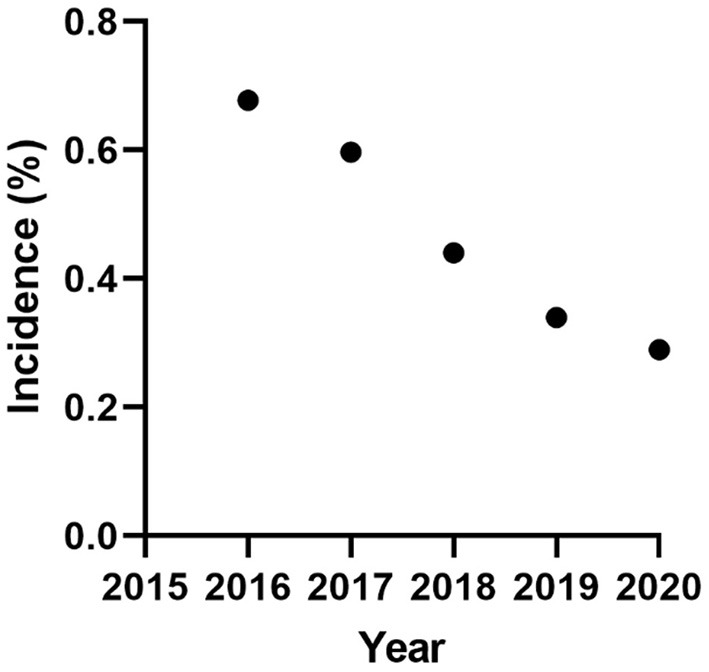
The annual incidence of animals disclosing as BVDv positive on the basis of their most recent test result.

**Figure 3:**
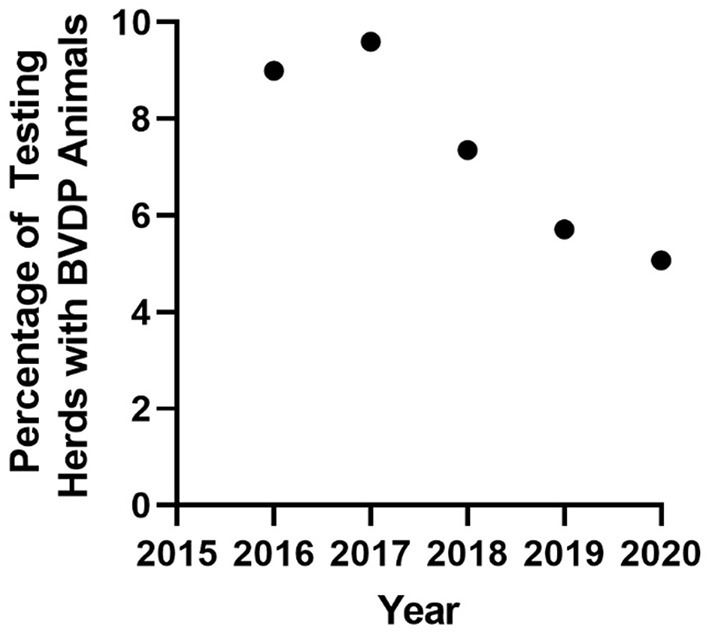
The annual incidence of testing herds with animals disclosing as BVDv positive (BVDP).

**Figure 4:**
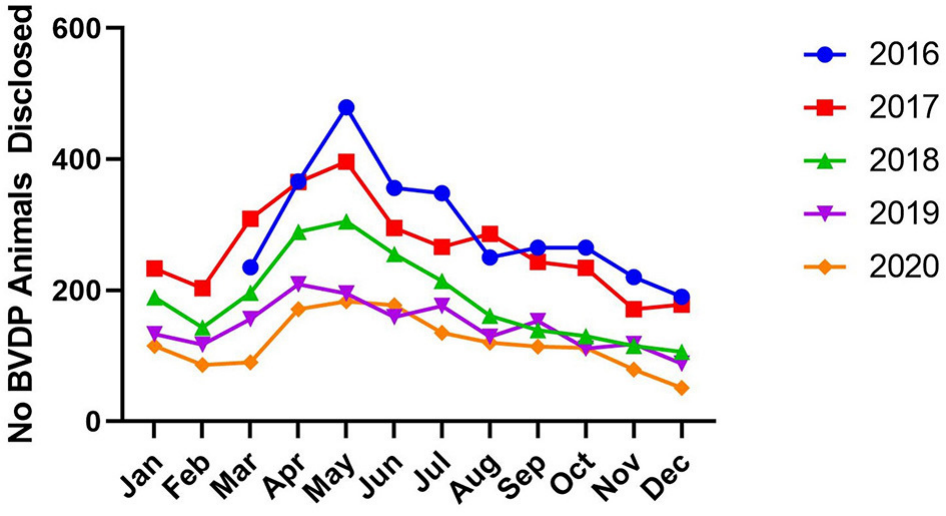
The monthly incidence of BVDv positive cattle (BVDP) disclosure during the compulsory phase of the BVD eradication programme.

Infection is distributed across NI, and the reduction of infection intensity has been evenly reduced across the province (Figure 1). The initial herd incidence (percentage of breeding herds in which BVDv positive animals were born between March and December 2016) was 0.68% and has reduced consistently year on year to an incidence of 0.29% for the full year of 2020 (Figure 2). Related to this, the percentage of testing herds that had BVDv positive animals has reduced from a peak of 11.3% for the period March 2016 to Feb 2017 to 5% in 2020 (Figure 3). A consistent seasonal pattern in peak BVDv incidence time has been observed during April and May, largely reflecting the peak in calf births within NI, as well as a consistent reduction in the number of BVDP animals detected each month of each year (Figure 4).

**Figure 5:**
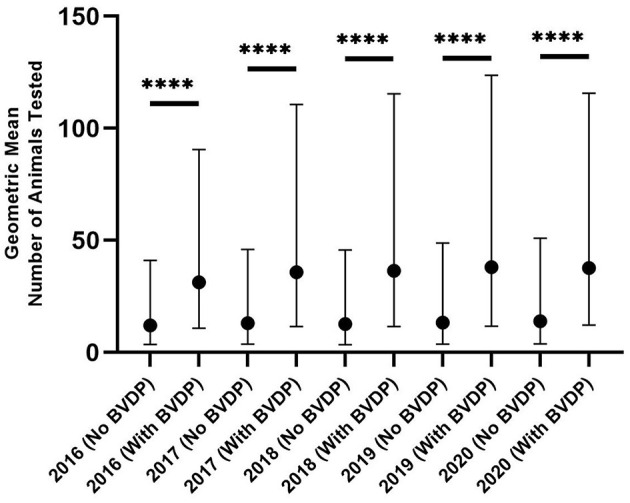
The number of animals tested per herd for herds that have and have not disclosed BVDP animals (^****^ *p* < 0.0001).

**Figure 6:**
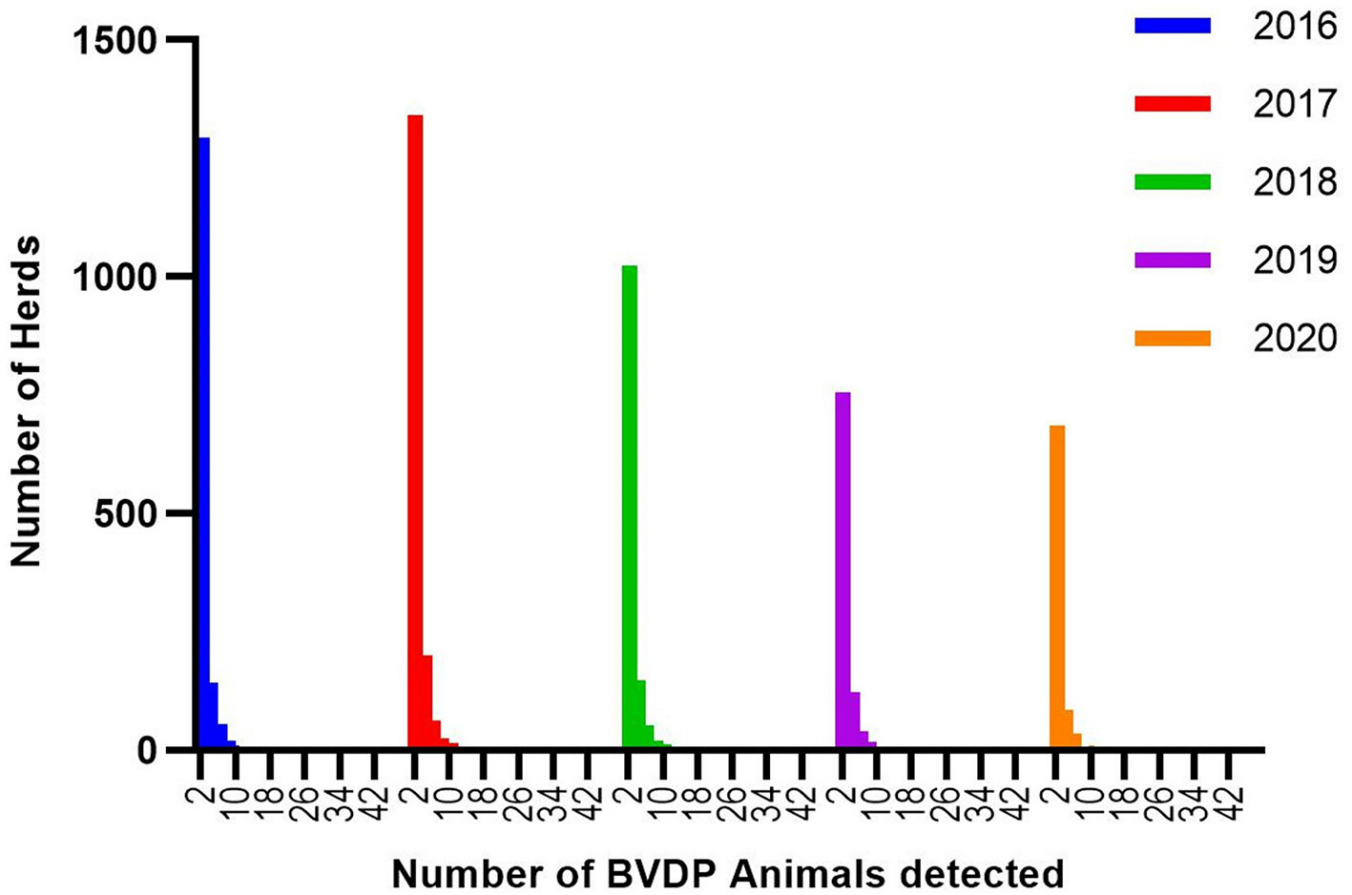
The distribution of BVDv positive (BVDP) animals disclosed in each herd annually.

Throughout the period of the compulsory programme there is a strongly significant association between the number of animals tested and the likelihood of BVDP animals being disclosed (*p* < 0.001 for each year using the Kolmogarov -Smirnov test) (Figure 5). Despite this, the great majority of herds with BVDP animals have four or fewer positive animals per year with the mode being one animal per herd (Figure 6) (range 1–47).

**Figure 7:**
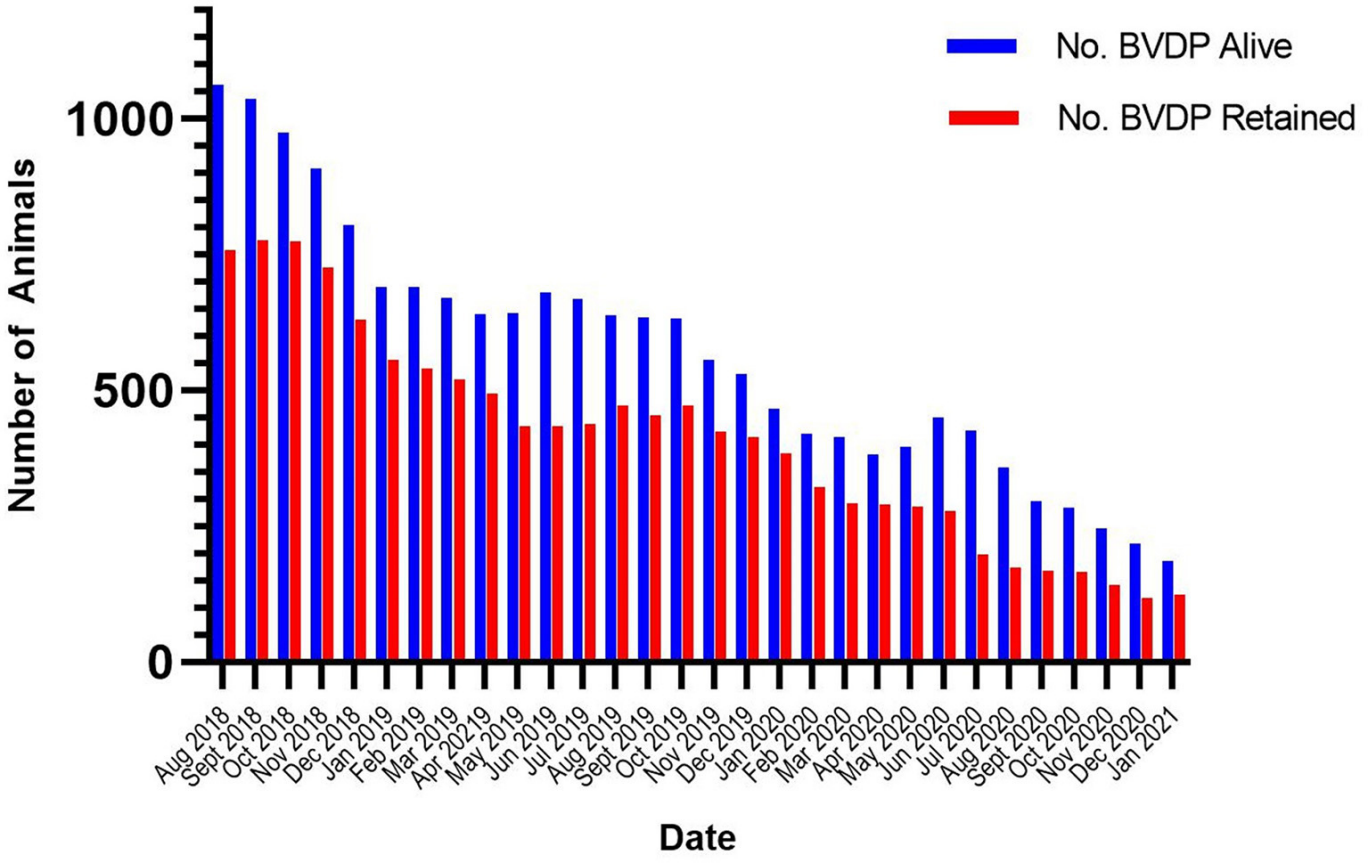
The number of all BVDv positive (BVDP) animals alive and the number of BVDP animals alive 35 days after disease status disclosure at the start of consecutive months from July 2018 to January 2021.

Overall, the number of BVDP animals that are retained have reduced considerably over time. From the point that this data was first recorded (June 2018) to the start of 2021, the number of all disclosed BVDP animals alive at the start of each month has reduced by 82% and the number of BVDP animals deemed as retained (i.e., still alive 35 days after disease status has been set) was reduced by 84% (Figure 7). However, it should be noted that this percentage reduction is a relative rather than an absolute figure as new cases are constantly emerging, albeit at a reduced rate. The continued emergence of cases of BVD in 2021 has provided evidence of the carryover of infection in herds from 2020. In the majority of cases, infection is found in herds with a recent history of infection disclosure strongly suggesting that infection can be attributed to the retention of BVD Positive (BVDP) cattle and the probable infection of susceptible females during the first to fourth months of pregnancy. For example, during January 2021, there were 96 individual cases of BVD disclosed in 70 herds. Of these herds, over three quarters (54 of the 70 herds) had BVDP animals disclosed during 2020.

All testing for the programme must be accredited to the ISO 17015 standard and the test results able to be uploaded in a pre-determined format to the AHWNI database. To comply with programme rules, labs must upload 95% of results within 7 working days and 99% within 10 working days from receipt of the samples. For the period 2016–2020, 98.8% of results were uploaded within 7 working days and 99.2% within 10 working days. The median test turnaround for each year was 2 days for 2016 and 1 day for 2017-2020.

### AHWNI Johne's Dairy Control Programme

Eighty two herds were included in the analysis. A summary of the key findings is made in Table 4. One third (14) of participating herds reported having suspect clinical cases of Johne's Disease present in the herd although only 18 (21%) reported having carried out a herd test. The most common risks identified in participating herds were: animal introductions, the use of contractors to spread slurry, mixing of cattle with other herds, feeding of whole milk and colostrum from cows other than the calf's dam, use of the calving pen for sick animals, failure to segregate high risk animals at calving and leaving calves with their dam.

**Table 4:** Summary of the Johne's disease risks identified using veterinary risk assessments carried out by AHWNI approved veterinarians from October 2020 To March 2021.

**Risk**	**Finding**
Introduced animals to the herd in the previous 5 years.	75 (89%)
Suspect Clinical Case/s.	27 (33%)
Use of contractors to spread slurry.	42 (50%)
Mixing of cattle with neighbouring herds.	39 (46%)
Contact with sheep.	31 (37%)
Colostrum from other cows with no selection on donor cow JD status.	13 (15%)
Calves fed whole milk from cows with no selection on JD status.	22 (26%)
Non-saleable milk fed to calves.	17 (20%)
Pre-weaned calves kept in groups of 9 or more.	13 (15%)
Calf exposure to adult cattle faeces.	8 (10%)
Manure above hocks and on flanks and udder of more than 10% of springing cows before entering the calving area.	8 (10%)
Manure above hocks and on flanks and udder of more than 10% of springing cows after entering the calving area.	9 (11%)
Visible manure covering some of the calving pen.	15 (18%)
Calving area used to house sick of lame cows at least every month.	19 (23%)
JD high risk cows including those showing clinical signs consistent with JD allowed to calf in the same area as other cows.	21 (25%)
>5% of cows calf in non-designated areas such as cubicle houses.	5 (6%)
>10% of calves allowed to suckle their dam.	5 (7%)
<10% of calves are removed from their dam within 30 min.	44 (52%)

### Cattle Health Scheme Results

AFBI CHS has 321 active members. Of these there are 138 herds with JD Risk Level 1 status, 48 herds AF for BVD, 76 herds VMF for BVD, 15 herds AF for IBR, 6 herds VMF for IBR, 11 herds Leptospirosis AF, 1 herd Leptospirosis MF and 6 Risk Level 1 for Neosporosis. AFBI not only tests CHS samples but diagnostic and surveillance samples from across NI farms. Table 5 shows the percentage of positive samples from diagnostic samples compared to those from AFBI CHS herds in 2019. It should be noted that diagnostic samples are likely to be taken from clinical cases and therefore have a higher chance of being positive.

**Table 5:** A summary of AFBI diagnostic and CHS results.

**Disease**	**Positive diagnostic samples**	**Positive AFBI CHS samples**
Johne's Ab	860/6222 (13.8%)	259/9454 (2.7%)
MAP PCR	274/1223 (22.4%)	10/709 (1.4%)
BVD Ab	308/726 (42.4%)	215/813 (26.4%)
BVD Ag	36/1163 (3.1%)	0/605 (0%)
IBR	502/964 (52.1%)	63/584 (10.8%)
IBR gE	31/191 (16.2%)	9/677 (1.3%)
Leptospirosis	206/369 (55.8%)	79/657 (12.0%)
Neosporosis	183/1394 (13.1%)	2/319 (0.6%)

*Data from 2020*.

## Discussion

The NI BVD Eradication Programme is delivered and managed by the not-for-profit company Animal Health and Welfare NI (AHWNI). The BVD programme is unique within NI as it is the only disease of livestock under legislative control where the management, delivery and direct funding of the programme is by the Agri-Food industry. The annual costs of the programme to the industry, taking account of testing and programme management, is in the region £1.2 million per year. The Programme works in partnership with DAERA who are responsible for the relevant legislation and its enforcement. It has been developed through a staged process, initially through a voluntary programme which demonstrated the technical ability of the industry to deliver such a programme and that the NI industry had sufficient appetite for a compulsory programme followed by the current legislated eradication programme. This phased approach to programme development through a voluntary phase followed by a compulsory phase is typical of many control programmes internationally (15). During 2015, 175,356 animals were tested which was 37% of all animals <1 year of age (13). This figure was important as in order to progress legislation for the statutory control of BVD, DAERA required evidence that there was sufficient appetite for legislative controls within the NI farming community. The threshold set was that more than 30% of the annual crop of animals born should be subject to voluntary control programme testing.

The annual rolling prevalence of BVD at the animal level has decreased by 57% since the end of the first 12 months of the compulsory programme from 0.68% to 0.29% by the end of 2020. The annual herd incidence of BVD in herds has decreased by 56% since the end of the first full year of the compulsory programme, from 11.46% to 5% at December 2020 representing a substantial level of success for the programme to date. The decrease in prevalence from the start of the compulsory programme in NI in March 2016 has occurred largely as a result of industry measures to deal with BVD positives, including voluntary culling of PIs by herd owners. No financial support to assist in the disposal of PI cattle has been made available to farmers by government or any other source, apart from the period February to September 2017 when modest support was provided under EU Exceptional Adjustment Aid (EAA) to farmers who were culling BVD Positives. Just under 1,000 claims were made. It should also be noted that other factors such as the concerted communication effort from AHWNI and increasing farmer awareness of BVD are also plausible contributory factors of the effects seen. However, while there is an overall pattern of successive decreases in animals and herd incidence year on year, there is some evidence of a slowing of progress given the relative reduction in incidence between 2019 and 2020 is less than that in previous years. This may suggest that the gains made through the industry-led measures may be reaching their limit and that additional enforcement activities will be necessary to allow further decreases and progress to eradication.

Historically in the Programme, many farmers made an active decision to keep their calves and “take the chance” that they could finish them for beef, as there were no overt scheme disincentives beyond the restriction and isolation requirements imposed legislatively on positive calves. However, the novel industry led programme developments of abattoirs voluntarily refusing to slaughter BVDP animals and the retention of BVDP animals as a non-conformance within the NI Farm Quality Assurance Programme have had a positive effect on influencing farmer behaviour as evidenced by a substantial reduction in the number of BVDP animals alive. However, while the overall numbers have reduced, the proportion of these animals that are retained, that is, still alive 35 days following disease status disclosure, has remained similar, indicating that retention of BVDP animals remains a substantial challenge to the programme. While it is possible that there may have been other sources of infection affecting premises with new BVDP disclosures, the picture being presented suggests that there are a significant number of cases where the BVD virus continues to circulate on farms largely due to the failure to cull PI calves in a proportion of herds. Certainly the evidence from other compulsory programmes strongly indicates the necessity of additional government led steps to influence herdowners to remove BVDP animals more quickly to achieve the ultimate eradication of the infection (16). For example the success of the BVD eradication programme in Norway was largely due to the collaborative approach between government and industry in that country (17, 18).

There is a strong and consistent association between the number of animals tested on each herd and the likelihood of finding positive animals. As testing is required for all new-born calves, this is a useful proxy for the size of breeding herds within NI. This association with herd size is well-recognised (19) and probably reflects an association with known infection risks such as animal movements, number of neighbour contacts and farm visitors. An earlier analysis of spatial and herd-level risk factors during the first year of the compulsory programme revealed BVD “hotspot” areas and showed that previous positive status, herd size and the number of positive neighbours within 4 km were positively associated with infection (20). Similarly, a risk factor analysis demonstrated that the risk of being a BVDv positive herd was positively associated with herd size, the numbers of births on farms and inward trade movements of cattle, calf mortality and number of tested animals (20).

Interestingly the distribution of positive animals has remained similar each year with the great majority of herds having 4 or fewer BVDP animals. It could have been speculated that as the level of circulating virus reduces, the proportion of susceptible animals might increase due to reduced prior exposure to the pathogen, thereby leading to an increasing number of BVDP animals in positive herds but this is not evidenced in the findings to date. This may be a reflection that there remains a substantial level of herd immunity within the cattle population and/or that there remains ongoing widespread vaccination against the infection. It could also reflect that the rapid removal of BVDP animals is managing to limit within herd spread. Importantly, given the modest number of animals removed each year, the likelihood of any detrimental effect on the genetics of the cattle population within Northern Ireland is very small.

There are a number of issues that remain to be resolved that are undoubtedly slowing the progress of the Programme. PI retention is believed to be the most important factor in the spread of BVDv (21). Measures to address the retention of PIs include the introduction of new legislation. Industry has made substantial voluntary efforts to drive programme change, however this has been limited to certain categories of farms (for example, those in the FQAS scheme). This need for novel governance measures to encourage compliance for programmes addressing non-zoonotic diseases has been highlighted elsewhere as has the need ultimately for “scaling up of responsibility from industry to government” (22). Interestingly modelling of various control programme scenarios in Germany suggests that tissue tag testing alone will be insufficient to eradicate infection in Germany (23). In the light of this, DAERA has agreed to progress new legislation, which the local Agri-Food industry is in support of, to provide additional controls that, it is hoped, will allow the programme to progress to infection eradication. This legislation will include restrictions on animal movement into and out of holdings with retained PIs, herd statuses, biosecurity notifications to herds neighbouring those herds with retained PIs, increased powers of enforcement and disease tracing. For example, there is currently no tracing facility available to the BVD Programme to allow tracing of dams that have potentially carried infection to new herds through their PI calves, having been in the window of susceptibility for infection before entering the herd in which they have calved. The ability to trace these so-called Trojan animals back to herds where infection may have taken place would allow the provision of tailored advice to infected herds as well as selling and purchasing herd owners. Legislation to allow the sharing of such data held by DAERA with the Programme would be of significant benefit. Progress in developing this legislation has been delayed due to other prioritisations within DAERA, the effects of Brexit and most recently the global SARS-CoV2 pandemic.

Under the provisions of the Northern Ireland Protocol of the Brexit Withdrawal deal, NI is obliged to align to the rules of the EU's Single Market, in areas such as technical regulation of goods, agricultural and environmental production and regulation. Therefore it is very likely that there will be a need to align the NI BVD Programme with the new EU Animal Health Law in order to avoid negative impacts on trade, in particular because of the progress that the Republic of Ireland BVD programme is making toward eradication (24). One benefit of this could be to address the current significant risk of reintroduction of BVD into NI through the movement of animals (25). At present there is no requirement for cattle being imported to NI from any other jurisdiction to have proof of a BVDv Negative test before entry, although cattle moving on to a holding that were born on or after March 1, 2016 must have a BVD test carried out within 20 days of coming into the control of a keeper. Provisions within the EU Animal Health Law, should they be applied to BVD controls within NI, would assist with mitigating this specific risk.

The proportion of herds participating in the other voluntary programmes is currently limited with less than 5% of eligible herds participating. This is mainly due to two factors. The great majority of participants in the Cattle Health Scheme programmes are pedigree beef herds where participation supports the sale of pedigree animals through the provision of animal and herd health declarations. The AHWNI JD Control Programme for dairy herds only commenced in October 2020 during the global SARS-CoV2 pandemic. Therefore, the number of herds participating in this programme at the date of writing has been limited. However this number is likely to substantially increase over the coming years due to recent changes to the UK Red Tractor Dairy Farm Quality Standard which requires all quality assured herds to participate in a Johne's Control Programme (14). Given that the majority of NI dairy herds are Red Tractor assured this will inevitably lead to a greater participation in the programme.

It is likely that those herds that have participated first in the programme are those most interested in the programme or have a perceived risk from Johne's Disease and so it may be that the findings to date do not represent NI dairy farms. Nonetheless it is interesting to note that many of the herds reported having substantial infection risks. The most common biosecurity risks observed were risk of introduction of infection, related to animal movements, the use of contractors to spread slurry and the mixing of cattle with other herds. These findings are consistent with other studies which have demonstrated the substantial risk of infection introduction to cattle herds in NI (3, 26).

The farmers also indicated the presence of substantial risks for infection establishment and spread. Most notably a large proportion reported the feeding of whole milk and colostrum from cows other than the calf's dam, the use of the calving pens for sick animals, the failure to segregate high risk animals at calving and leaving calves with their dam. All of these have been identified as potential risks for Johne's Disease transmission (27). While it is clear that a number of important risks are present on these farms, it is noteworthy that the farmers that participated were prepared to identify and acknowledge suspicion of infection and infection risks during the risk assessment process given the perceived stigma that can be associated with this infection (28). An important future outcome from the programme will be to measure progress in reducing those risks identified on participating farms.

The AFBI CHS offers a route for herds to remove endemic diseases from their herd and offers accredited statues for the diseases when the herd reaches the requirements of the disease programme. Herds with accredited statuses for the AFBI CHS diseases can promote their herds as having a high health status. Members of the control programmes are required to abide by the rules of the scheme as defined by the Cattle Health Certification Standards (CHeCs). Actions which are considered high risk and that compromise the health of the herd could result in a herd losing its accredited status. For example, two of the more common causes of this include failure to test added animals and not maintaining added animals in isolation facilities appropriately.

The number of accredited herds for IBR, Leptospirosis and Neosporosis is much lower than the number of accredited herds for Johne's Disease and BVD. Participation in the Johne's Disease programme may be higher as some breed societies have a requirement that animals attending sales are from herds with a Johne's Disease accredited status. BVD is also likely to have more accredited herds due to the NI BVD Eradication Programme. Herds already performing BVD testing of their calves can use the same results to gain an accredited status. A herd can therefore gain a BVD status at little or no additional cost if they do not buy in animals or have animals returning to the herd.

The reasons for fewer herds engaging with the IBR, Leptospirosis and Neosporosis programme may be due to the more challenging nature of these programmes or that there are more attractive alternatives to some herdowners. For example, many herds may choose to vaccinate against Leptospirosis which for some herdowners may be perceived to be cheaper (as leptospirosis vaccines are inexpensive) and a safer option than not vaccinating and demonstrating the herd to be serologically negative. Serologically negative herds will be susceptible to significant infection outbreaks and so some herdowners may perceive the risk of participating in the leptospirosis programme as a higher risk than simply vaccinating.

At times of economic hardship, continuing in a health scheme may seem like an unnecessary expense (29). Stopping membership and testing may lead to the farm having a short-term saving in money (membership fees and animal testing). However, in such a circumstance any disease statuses would be lost which may have been built up over several years, but more importantly the herd will likely be at higher risk of introducing disease into the herd if they are not following the biosecurity and added/returning animal rules. Therefore, there is a continual onus on programme providers, veterinarians, and industry leaders to highlight the value of participation in well-managed and designed control programmes. One of the advantages of any control programme is the potential for it to facilitate risk-based trading. Herds are encouraged to remain closed and to avoid buying in animals. However, in regions where there are high levels of animal movements such as NI, the need for purchasers to assess the infection risk of purchased animals is considerable. For example, farmers should be wary when considering purchasing an animal from a herd with unknown or high JD risk level. While most herds are not currently participating in the AFBI JD CHS, those that are, are largely pedigree herds selling stock bulls. Therefore, herds that are purchasing breeding bulls are advised to look for JD CHS low risk herds. Importantly the disease control programmes described here provide a valuable model for the design of risk-based trading systems for other diseases including those regulated under national or international laws.

In conclusion, this paper describes the range of control programmes for those infections of cattle that have not historically been subject to regulated controls. They demonstrate a range of approaches to disease control. The AFBI CHS programmes focus on providing individual herd level infection assurance and the AHWNI Programmes focus on control or eradication at the regional or sectoral (dairy) level. The formation of AHWNI by the Northern Ireland Agri-Food industry was a crucial step in progressing region wide disease control programmes. The success of the BVD eradication programme has demonstrated the ability of industry to take ownership of disease control programmes and to substantially reduce the incidence of infection using industry measures. However, it has also demonstrated the essential role government has in facilitating the ultimate eradication of infection from a region or country.

## Data Availability Statement

The raw data supporting the conclusions of this article will be made available by the authors, without undue reservation.

## Author Contributions

SS collated and analysed the data. SS, SV, and EC wrote the first draft of the manuscript. All authors contributed to conception of the study, manuscript revision, read, and approved the submitted version.

## Conflict of Interest

The authors declare that the research was conducted in the absence of any commercial or financial relationships that could be construed as a potential conflict of interest.

## Publisher's Note

All claims expressed in this article are solely those of the authors and do not necessarily represent those of their affiliated organizations, or those of the publisher, the editors and the reviewers. Any product that may be evaluated in this article, or claim that may be made by its manufacturer, is not guaranteed or endorsed by the publisher.

## Footnotes

^1^Available online at: https://www.lmcni.com/site/wp-content/uploads/2018/04/18-04-17-FQAS-beef-at-slaughter-1992-2017.pdf (accessed March 23, 2021).
