# Supplementary material for: The Northern Ireland Control Programmes for Infectious Cattle Diseases Not Regulated by the EU
Source: Front Vet Sci. 2021 Aug 26;8:694197. doi: 10.3389/fvets.2021.694197 (PMC8427759; doi:10.3389/fvets.2021.694197)
Supplement: Supplementary file 1 [file Table_1.DOCX]

**Criteria for Approval of Laboratories to Provide Tests in Support of the Northern Ireland BVD Eradication Programme.**

Approval will be on a test by test (e.g., detection of virus by ELISA or RTPCR) and matrix by matrix (e.g., tissue punch or blood) basis. In order to be approved, laboratories must confirm that they comply with the following:

1. Must have accreditation to ISO17025 for all relevant BVD tests (virus detection by RTPCR or ELISA and/or antibody detection by ELISA) for which DAERA approval is being sought. Documentary evidence must be provided to accompany the Application Form to DAERA and DAERA must be informed of any change in accreditation status as soon as practically possible, including any refusal of an application made by the laboratory for accreditation.
2. Participate to the satisfaction of DAERA in such relevant proficiency testing and technical reviews as may be considered appropriate.
3. Provide such data in relation to laboratory function, diagnostic testing and results as may reasonably be requested by DAERA to specified deadlines.
4. Facilitate such laboratory visits as may reasonably be requested by DAERA within specified deadlines.
5. Retain a record of all testing carried out in relation to the programme for at least 7 years and furnish copies/extracts from time to time to DAERA on request in such manner as DAERA may require.
6. Report results for all herds, where necessary permissions are in place, by electronic transfer at least once per day [day of report] to the AHWNI database in the following format or in such revised format as DAERA may from time to time specify:
   1. Herd number
   2. Lab delivery date (date of receipt)
   3. Test date
   4. Animal ID
   5. Individual lab reference
   6. Test name
   7. Sample type
   8. Test value
   9. Laboratory interpretation of that result
7. Provide test results within specified turnaround times (all measured on a monthly basis):
8. a.95% of results within 7 working days of receipt;
9. b.99% within 10 working days of receipt.
10. c. A median interval of 5 days or less
11. Maintain a structural error rate (as defined by DAERA) not exceeding 5% in data files transferred to the AHWNI database, measured on a monthly basis.
12. Demonstrate evidence, to the satisfaction of DAERA, of a viable contingency/emergency plan.
13. Retain and preserve samples for a period of 60 days from the date of the test.
14. Must log individually and electronically all samples received for BVDV testing under then NI BVD Eradication Scheme by the end of the next working day following their receipt by the laboratory.
